# Neurological features of children with food allergies

**DOI:** 10.1186/2045-7022-1-S1-P85

**Published:** 2011-08-12

**Authors:** Sam Lingam

**Affiliations:** 1 Harley Street Paediartic Chambers, London, UK

## 

Food allergy is a multisystem disorder. It presents with various neurological manifestations.

In a clinical study of 25 children attending a food allergy clinic the following neurological features were detected:

Irritability 25 (100%)

Behaviour problem18 (72%)

Hyperactivity 16 (64%)

Migraine 13 (52%)

Fatigue 13 (52%)

Clumsiness 4 (16%)

Over the years clinical practice also showed that the diagnosis of food allergy and appropriate management gives excellent symptom relief with tremendous job satisfaction. Parents appreciate the diagnosis and management when symptoms improve.

Recalcitrant eczema and hyperactivity in children could be due to food allergies. Abnormalities of behaviors and mood are not all in the mind; it can be improved by dietary manipulation.

## Conclusion

Food allergy is here to stay; clinicians should make the diagnosis by appropriate history, examination looking for clinical features and behaviour phenotypes and by investigations. A paediatric history questionnaire is helpful in obtaining the detailed allergy history. The questionnaire will be circulated to participants with the handout.

